# Totally thoracoscopic atrial fibrillation surgery following massive small bowel resection due to superior mesenteric artery embolization: report of two cases

**DOI:** 10.1186/s40792-024-01938-2

**Published:** 2024-06-11

**Authors:** Taisuke Nakayama, Yoshitsugu Nakamura, Kusumi Niitsuma, Masaki Ushijima, Yuto Yasumoto, Miho Kuroda, Kosuke Nakamae, Naoshi Minamidate, Yujiro Hayashi, Ryo Tsuruta, Yujiro Ito, Akira Furutachi, Hiroaki Yusa

**Affiliations:** Department of Cardiovascular Surgery, Chiba-Nishi General Hospital, 107-1 Kanegasaku Matsudo-Shi, Chiba, Chiba-Ken 270-2251 Japan

**Keywords:** Non-valvular atrial fibrillation, Superior mesenteric artery occlusion, Short bowel syndrome, Left appendage closure, Pulmonary vein isolation, Thoracoscopic surgery

## Abstract

**Background:**

Thromboembolic occlusion of the superior mesenteric artery (SMA) is a grave complication in individuals diagnosed with atrial fibrillation (AF). This condition often necessitates extensive bowel resection, culminating in short bowel syndrome, which presents challenges for anticoagulant administration and/or antiarrhythmic therapy.

**Case presentation:**

Presented here are findings of two patients, aged 78 and 72 years, respectively, who underwent comprehensive thoracoscopic AF surgery subsequent to extensive small bowel resection following SMA embolization. In each, onset of AF precipitated an embolic event, while the concurrent presence of short bowel syndrome complicated anticoagulation management. Total thoracoscopic AF surgery, comprised stapler-closure of the left atrial appendage (LAA) and bilateral epicardial clamp-isolation of the pulmonary veins, an operative modality aimed at addressing AF rhythm control and mitigating embolic events such as cerebral infarction, led to favorable outcomes in both cases. Additionally, computed tomography (CT) conducted one month post-surgery revealed the absence of residual tissue in the LAA, with the left atrium demonstrating a well-rounded, spherical shape. At the time of writing, the patients have remained asymptomatic following surgery regarding thromboembolic and arrhythmic manifestations for 29 and 10 months, respectively, notwithstanding the absence of anticoagulant or antiarrhythmic pharmacotherapy. Additionally, electrocardiographic surveillance has revealed persistent sinus rhythm.

**Conclusions:**

The present findings underscore the feasibility and efficacy of a total thoracoscopic AF surgery procedure for patients presented with short bowel syndrome complicating SMA embolization, thus warranting consideration for its broader clinical application.

## Background

Acute occlusion of the superior mesenteric artery (SMA) can lead to diffuse bowel damage, necessitating extensive bowel resection in severe cases [1]. Short bowel syndrome (SBS) can have significant effects on nutrient and medication absorption, including essential drugs such as anticoagulants, particularly in patients with atrial fibrillation (AF) [2].

Presented here are clinical experiences with thoracoscopic closure of the left atrial appendage (LAA) and bilateral epicardial isolation of the pulmonary vein (PV) in two patients affected by symptomatic persistent AF. Both had SBS complicated by thromboembolic occlusion of the SMA, leading to small intestine resection, thus rendering them unable to receive prophylactic anticoagulation or antiarrhythmic medication.

## Case presentation

### Case 1

**Fig. 1:**
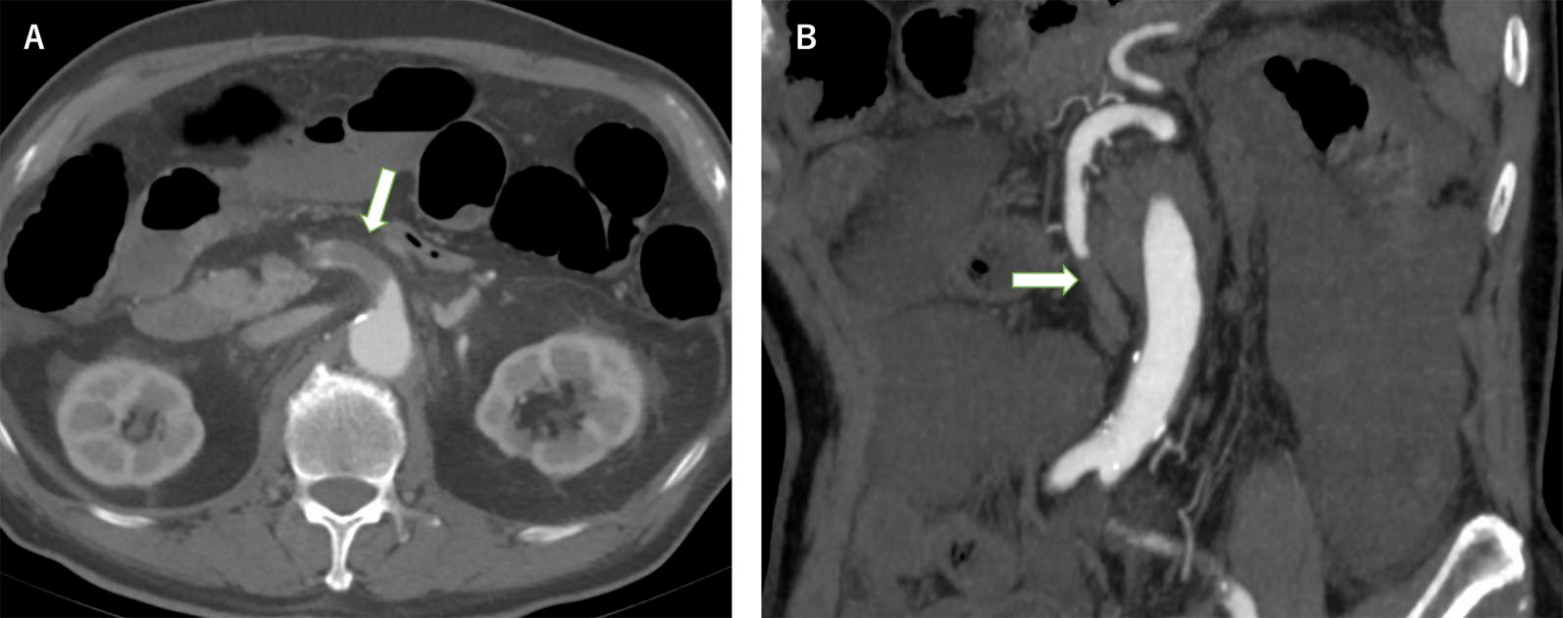
Contrast-enhanced computed tomography scanning revealed total occlusion of the superior mesenteric artery (arrows) (**A** Case 1, **B** Case 2)

A 78-year-old male was presented with severe abdominal pain, accompanied by vomiting and diarrhea, prompting urgent transfer to another medical facility. He had a history of atrial fibrillation; however, it went unnoticed, and he was not receiving the required anticoagulation therapy. The ECG findings upon admission indicated characteristics consistent with long-standing persistent atrial fibrillation. Enhanced computed tomography (CT) revealed disrupted blood flow originating from the SMA ostium (Fig. 1A). Emergency thrombectomy of the SMA, along with partial resection of the small intestine and cecum, were performed on the day of admission. A second-look surgical procedure was then done the following day, which included more extensive mass resection of the small intestine and also resection of the right hemicolon. One month later, the patient experienced gallbladder perforation, necessitating a cholecystectomy and open drainage. Challenges arose concerning drug absorption due to the extensive bowel resection, complicating initiation of warfarin therapy for anticoagulation. Consequently, there was an increased risk of reinfarction and bleeding due to inadequate anticoagulation control. Given the complexities associated with effective anticoagulation for the patient, therapeutic intervention for the LAA was deemed necessary.

**Fig. 2:**
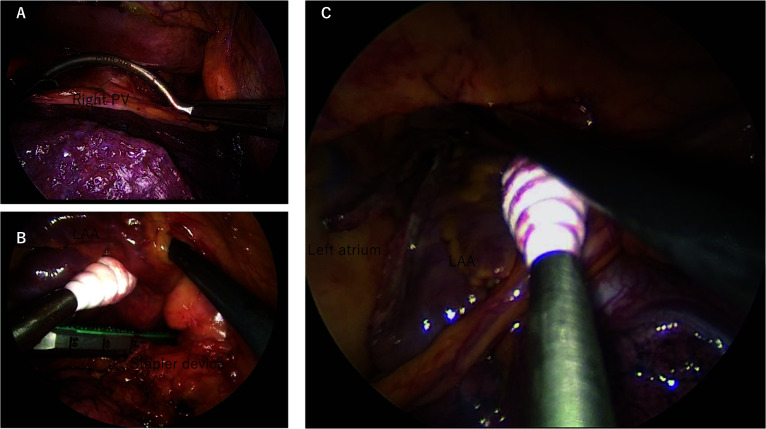
Intraoperative images from Case 1. **A** Pulmonary vein isolation was accomplished utilizing radiofrequency bipolar epicardial coagulators, **B** while closure of the LAA was achieved utilizing an automatic cut-and-staple device. **C** The transected surface of the LAA was straight

The patient was later referred to our institution for thoracoscopic surgery to manage the LAA and for rhythm control. The procedure was performed three months after the bowel resection. Preoperative echocardiography showed preserved left ventricular function (ejection fraction 60%), mild mitral regurgitation, and no significant valvular abnormalities. With the patient in a supine position, general anesthesia was administered utilizing a double-lumen endotracheal tube to facilitate hemipulmonary collapse. Four ports were inserted through appropriate intercostal spaces employing a technique previously described by Otsuka et al. [3]. Pulmonary vein isolation was accomplished utilizing radiofrequency bipolar epicardial coagulators (Isolator Synergy Clamps and Isolator Transpolar Pen, Atricure, USA) (Fig. 2A), while closure of the LAA was achieved utilizing an automatic cut-and-staple device (ECHELON FLEX Powered ENDOPATH Stapler 60, ETHICON, USA) (Fig. 2B, C). The surgical approach was commenced on the left side to effect LAA closure and isolate the left PV, followed by the right side for isolation of the right PVs. Intraoperative transesophageal echocardiography confirmed satisfactory closure of the LAA. The procedure was successfully completed without complications in 95 min.

**Fig. 3:**
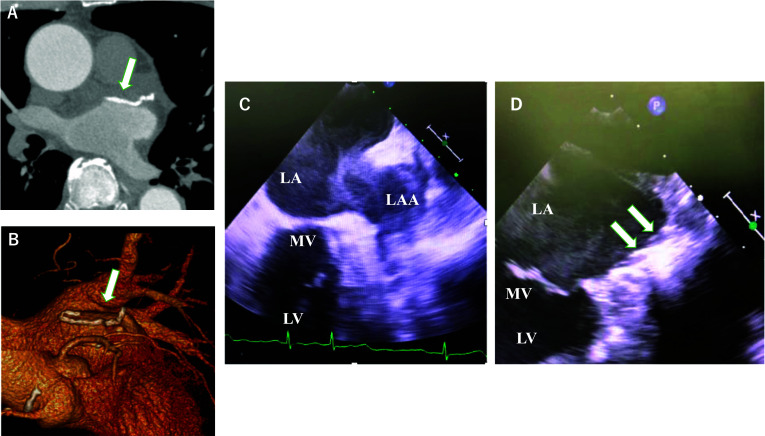
Post-operative contrast-enhanced computed tomographic images. **A, B** Case 1. The LAA was resected from the base (arrows). **C, D** Case 2. Transesophageal echocardiograph images obtained before (**C**) and after (**D**) left atrial appendage closure. *LA* left atrium, *LV* left ventricle, *MV* mitral valve

Following surgery, warfarin was administered for three weeks to maintain the prothrombin time-international normalized ratio (PT-INR) within a range of 1.5–2.0, after which it was discontinued. At one month following surgery, enhanced CT revealed the absence of clot formation within the left atrium (Fig. 3A, B). At the time of writing, the patient has remained asymptomatic regarding thromboembolic and arrhythmic manifestations for 29 months following surgery, despite the absence of anticoagulant or antiarrhythmic medication. Electrocardiographic monitoring has consistently demonstrated a stable sinus rhythm.

### Case 2

A 72-year-old man initially presented with long-standing persistent atrial fibrillation. However, due to a CHADS2 score of 1, anticoagulation therapy was not initiated during the follow-up period. He was later admitted to our hospital with severe abdominal pain and significant diarrhea. Physical examination findings indicated significant muscle tone consistent with the presence of panperitonitis. Emergency enhanced CT revealed complete occlusion of the SMA trunk (Fig. 1B), while continuous AF was evident in electrocardiography results. The diagnosis was acute thromboembolic occlusion of the SMA, attributed to persistent non-valvular AF. Emergency total intestinal resection from the jejunum to the ileum and a right hemicolectomy were performed due to extensive small intestine necrosis from the ligament Treitz and multiple intestinal perforations.

The patient was subsequently referred to our department for thoracoscopic surgery aimed at LAA management and rhythm control. Preoperative echocardiography indicated satisfactory cardiac function (EF > 60%), mild left atrial enlargement, with mitral regurgitation noted to be less than mild, and no other significant valvular abnormalities were observed. The surgery was performed 4 months after bowel resection. Utilizing the same techniques as described for Case 1, LAA resection and bilateral pulmonary vein isolation were performed. Intraoperative transesophageal echocardiography demonstrated disappearance of moyamoya echoes flowing from the LAA into the left atrium following LAA resection, along with a reduction in moyamoya echoes within the left atrium (Fig. 3C, D). The procedure was completed without complications in 96 min.

Post-operative echocardiography confirmed successful resection of the LAA at its root, with no thrombus detected in the left atrium. In concordance with Case 1, a distinct and cleanly dissected surface of the left atrium was observed using postoperative contrast-enhanced CT imaging. Warfarin was administered for one month postoperatively, after which it was discontinued following dose reduction. Similar to Case 1, at postoperative 10 months, the patient had not received anticoagulant or antiarrhythmic medications, nor reported thromboembolic or arrhythmic symptoms. Additionally, electrocardiographic monitoring at three months following surgery revealed a stable sinus rhythm.

## Discussion

SMA occlusion is a condition characterized by obstruction of the SMA by an embolus or thrombus, leading to extensive intestinal ischemia affecting both the small and large intestines. Unless promptly treated, SMA occlusion carries a high mortality rate [1]. Thrombosis leading to SMA occlusion may arise from emboli originating from the heart, such as the LAA, or from atherosclerosis or stenosis of the mesenteric artery [2]. Despite advancements in medical management for facilitating early diagnosis and intervention, the mortality rate of SMA occlusion remains alarmingly high, ranging from 60 to 80%, thus it is associated with a generally poor prognosis [4, 5].

Presently, a range of anticoagulant options, including direct oral anticoagulant (DOAC) administration, is available for prevention of thromboembolism in patients with AF. However, absorption of anticoagulants primarily occurs in the stomach and proximal small intestine [6–8]. Following extensive resection of the small intestine, notable structural and functional changes are known to occur in the remaining intestinal tract, including dilatation, wall thickening, alterations in villi morphology, and increased expression of sodium–glucose cotransporter 1 (SGLT-1) [9]. Such alterations can significantly impact drug absorption, including anticoagulants. Additionally, many of the anticoagulants available, including DOACs, lack easily accessible methods for monitoring in the bloodstream.

Buchholz et al. [10] reported that a functional small bowel length of 60 cm or more is advantageous for use of rivaroxaban. However, even a vitamin K antagonist such as warfarin, which can be monitored, poses challenges for achieving therapeutic control due to absorption issues. Therefore, when devising a medication regimen, careful considerations regarding overall health and nutritional status of the patient, as well as laboratory parameters, treatment efficacy, potential side effects, and other pertinent factors are imperative. Fortunately, both of the present patients had greater than 100 cm of the small intestine remaining. Nevertheless, they were subsequently presented with compromised renal function, necessitating management with warfarin, though their anticoagulation monitoring values remained markedly unstable despite treatment.

Given the aforementioned challenges, discontinuation of anticoagulation therapy in both cases became necessary, thus necessitating meticulous and dependable management of the LAA. In 2003, thoracoscopic left atrial appendage resection emerged as a non-pharmacological intervention procedure for left atrial thrombus. This technique effectively mitigates the risk of embolism in patients with non-valvular AF in the short term, with no observed neurological complications [11]. Furthermore, a report by Ohtsuka et al. presented a left thoracoscopic stapler left atrial appendage resection method that facilitates stapler insertion at an optimal angle, resulting in a precise dissection plane [12]. This approach permits additional loop procedures on the dissected surface, rendering it as an optimal technique for achieving comprehensive left atrial appendage management.

In the present two patients, transcutaneous catheter-based ablation was deemed inappropriate for two primary reasons. Firstly, anticoagulation is necessary post-catheter ablation until endothelial lesions have healed [13]. Conversely, the technique employed in these cases is known to rapidly isolate the PV from the epicardial side, thus minimizing endothelial damage [14]. Second, while AF may recur, affected patients face renewed risk of thromboembolism, which is particularly heightened with advancing age. Although AF recurrence remains a possibility in our patients, the continued low thromboembolic risk is attributed to removal of the LAA. While transcutaneous implantation of an LAA-closure device, such as Watchman or Amplatzer, might have been considered, the present straightforward cutting method was deemed preferable due to reported instances of clot formation on such devices, thus necessitating ongoing anticoagulant therapy [15, 16]. Despite this, both patients received warfarin treatment for 3–4 weeks. The procedure involved temporary myocardial blockade, such as pulmonary vein isolation with Atricure and left ventricular closure with Stapler. While minimal endocardial damage may occur, inflammation at the site is expected to persist. Indeed, Inoue et al. [17] reported a 15.8% incidence of thrombus formation near the LAA during the acute phase of LAA closure using a stapler. Although the clinical significance remains unclear, our department follows a protocol of administering empirical anticoagulation for approximately one month until contrast-enhanced CT confirms a thrombus-free closure area in the left atrium, as we routinely perform. The protocol essentially dictates discontinuing anticoagulation after 1 month, regardless of residual atrial fibrillation, upon obtaining contrast-enhanced CT images indicating a clear absence of thrombus, as demonstrated in the two patients. Further evidence in this area is warranted. In this context, thoracoscopic left atrial appendage closure emerges as an exceptionally favorable procedure, offering robust closure management and facilitating the gradual cessation of anticoagulation therapy. In 2018, a total of 201 patients in Japan underwent thoracoscopic LAA resection (Wolf–Ohtsuka procedure) [18]. Over a mean follow-up period of 48 months (range 12–110 months) after completion of anticoagulation therapy, only two developed cardiogenic thromboembolisms, translating to 0.25 events per 100 patient-years. Based on those findings, we propose this method as a viable option for preventing recurrence of ischemic stroke in patients with left atrial appendage thrombus refractory to medical therapy. Despite its invasiveness and the necessity of general anesthesia in contrast to other LAA management techniques, the procedure offers the advantages of a small incision (eliminating the need for cardiopulmonary bypass) and a brief operative duration. We propose this approach as a viable strategy for mitigating the risk of recurrent ischemic strokes in such patients.

In the two cases presented here, we were fortunate enough to successfully restore the patients to sinus rhythm through pulmonary vein isolation alone. Although pulmonary vein isolation is typically effective for paroxysmal atrial fibrillation, its efficacy diminishes for persistent cases, where extensive myocardial ablation via the maze procedure, as advocated by Cox et al. [19], proves superior. However, in the context of the two cases under study, the primary objective is the prevention of embolism and the gradual discontinuation of anticoagulant therapy through LAA management, with achieving perfect sinus rhythm being of secondary importance. Moreover, the invasiveness associated with a full maze procedure utilizing artificial heart–lung support would be more than twice as high, prompting the present study to confine its intervention to rhythm therapy within the scope of thoracoscopic manipulation. Another advantage of this treatment is its flexibility. In the event of atrial fibrillation recurrence, a hybrid approach is available, permitting a second attempt at rhythm therapy through supplementary catheter ablation. In these two cases, we were fortunate to achieve sinus rhythm solely through bilateral pulmonary vein isolation. However, if maintaining sinus rhythm became problematic, we had a plan to explore the option of additional catheter ablation postoperatively.

## Conclusion

The present results underscore the potential of the thoracoscopic technique utilized, which incorporates stapler-closure of the LAA and bilateral epicardial clamp-isolation of the pulmonary veins, as a viable treatment modality for patients presented with symptomatic non-valvular AF and analogous complications, such as those encountered in these two cases.

## Data Availability

There are no additional data to disclose.

## Abbreviations

SMA: Superior mesenteric artery
SBS: Short bowel syndrome
AF: Atrial fibrillation
LAA: Left atrial appendage
PV: Pulmonary vein
DOAC: Direct oral anticoagulants
